# The Anti-Apoptotic Role of Neuroglobin 

**DOI:** 10.3390/cells1041133

**Published:** 2012-11-23

**Authors:** Thomas Brittain

**Affiliations:** School of Biological Sciences, Centre for Brain Research, University of Auckland, 3a Symonds Street, Auckland,1142, New Zealand; Email: T.Britain@auckland.ac.nz; Tel.: +64-9-373-7599; Fax: +64-9-373-7414

**Keywords:** apoptosis, neuroglobin, neurons

## Abstract

The small heme-protein neuroglobin is expressed at high concentrations in certain brain neurons and in the rod cells of the retina. This paper reviews the many studies which have recently identified a protective role for neuroglobin, in a wide range of situations involving apoptotic cell death. The origins of this protective mechanism are discussed in terms of both experimental results and computational modeling of the intrinsic pathway of apoptosis, which shows that neuroglobin can intervene in this process by a reaction with released mitochondrial cytochrome c. An integrated model, based on the various molecular actions of both neuroglobin and cytochrome c, is developed, which accounts for the cellular distribution of neuroglobin.

## 1. Introduction: Neuroglobin Discovery and Basic Characteristics

The heme proteins hemoglobin and myoglobin are probably the best characterized proteins known. This situation is of course, in the main, due to their very high abundance and highly intense colour, together with their ease of isolation. Hemoglobin and myoglobin were the first proteins to have their structure determined and their functions in terms of oxygen and carbon dioxide transport, in the case of hemoglobin, and facilitated oxygen diffusion, in the case of myoglobin, are well determined. For many decades it was assumed that these two proteins represented the only two heme containing globins in the human body. It was not until 2000 that neuroglobin was identified as a third heme containing globin in the human. Its discovery was a paradigm of modern molecular biology. Burmester and colleagues [1] searched the newly published human genome to explore the possible existence of other heme containing globins, using well known amino acid sequence signatures associated with these proteins. They discovered a putative heme containing globin gene on chromosome 14q24 between markers DI 4576 and WI 4643 and were soon able to show that this gene was indeed transcribed and translated into a protein which could be identified in the human brain neurons—hence the newly discovered proteins name—Neuroglobin. The neuroglobin gene has been shown to consist of a unique globin exon/intron structure with 5 exons and 4 introns, but never the less shows the classical 3 on 3 globin fold [2,3,4]. Recently, expression control elements have been identified upstream of the structural gene [5]. Extensive genetic and evolutionary studies have shown that neuroglobin is a very ancient protein, sharing a last common ancestor with myoglobin and hemoglobin branches 800 million year ago [6]. The protein is very highly conserved, showing 94% identity between human and mouse [7]. It also shows close homology with the nerve globins of invertebrates [8] but little identity with either myoglobin or hemoglobin.

**Figure 1:**
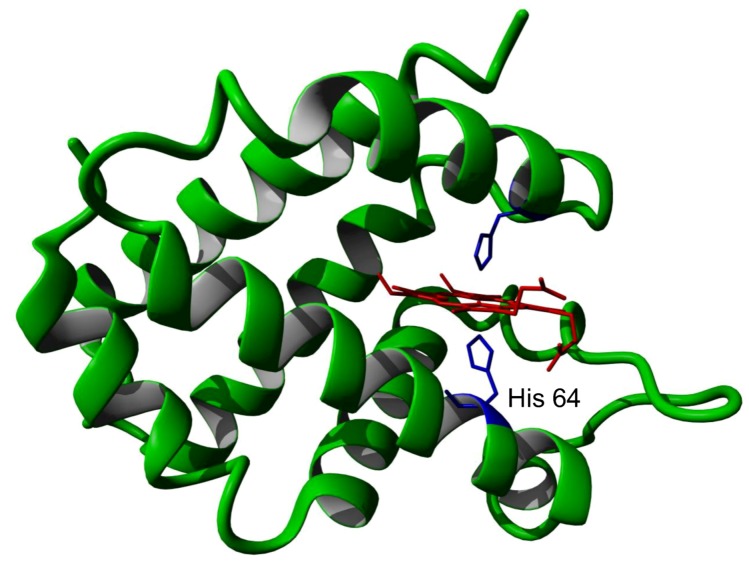
Crystal structure of human neuroglobin showing the heme group (red) and iron bound histidine side chains (blue). The partially dissociated histidine 64 is indicated.

Following its initial identification, methods were rapidly developed for the production of large quantities of recombinant protein produced in *E. coli* [9]. A number of groups then determined the detailed physico-chemical properties of this new protein. The protein consists of a single polypeptide chain containing 151 amino acids, yielding a mature protein of 17 kDa molecular weight with a very acidic isoelectric point of 4.6. The structure of the mouse and human proteins have been determined at high resolution and shown to contain a protoporphyrin IX heme group held in the protein by coordination of the heme iron to two histidine amino acid side chains (Figure 1). 

It should be noted that the close similarity of the reported ferrous and ferric forms of the protein might well arise from the X-ray beam induced photo-reduction of the ferric form, such that both the reported ‘ferric’ and ‘ferrous’ forms may well both be in fact the ferrous form [2,3,4,10]. One of the heme ligand histidine residues (His 64) is relatively weakly bound the heme iron atom such that the protein can bind the common heme ligands oxygen, carbon monoxide, NO *etc.* This exchange of heme iron ligands in neuroglobin which requires the initial loss of the natural heme ligand produces a protein which has both slower binding rates and weaker binding affinity than would otherwise be expected [11,12,13,14,15]. Another unexpected characteristic of neuroglobin is its extreme pH and temperature insensitivity. It is reversibly denatured only at pH values as low as 2.0 and a temperature of greater than 100 °C [16,17,18]. The heme iron can undergo a simple one electron reduction from the ferric to ferrous forms with an associated redox potential of −129 mV [12].

## 2. Normal Expression and Distribution

Neuroglobin is expressed in neurons, retinal cells and some endocrine tissues and is apparently present in all vertebrates. As such, it is generally associated with tissues which exhibit the highest oxygen consumption rates and poor if any capacity to replicate [19]. In brain tissue as a whole neuroglobin is present at approximately 1 μM [20]. The earliest studies of the distribution of neuroglobin expression in brain tissue gave some conflicting results. Immunocytochemistry, employing polyclonal antibodies, indicated the presence of neuroglobin in the neurons of the medial vestibular nucleus and paraolivary nucleus and to a lesser extent the thalamic and subthalamic regions of the mouse brain [21]. *In situ* hybridization and RT-PCR indicated neuroglobin expression in focal regions of the mouse brain, under normoxic conditions, including the lateral tegmental nucleus, preoptic nucleus, amygdale, locus coeruleus and the nucleus of the solitary tract [22]. In contrast *in situ* hybridization results were initially interpreted as showing a uniform distribution of neuroglobin expression [23]. In 2003 the same research group, using immunohistochemistry techniques with a polyclonal antibody to the 55–70 fragment of neuroglobin identified differential neuroglobin expression in various regions of the mouse brain, with significantly high expression in the cerebral cortical regions, thalamus, hypothalamus and nuclei of the cranial nerves in the brainstem and cerebellum [24]. They later showed that the neuroglobin reactivity was associated with neuronal but not glial cells [25].

In a recent series of papers Hundahl and colleagues used *in situ* hybridization, immunochemistry and immunoelectron microscopy to show high levels of neuroglobin in the piriform complex amygdala, hypothalamus, medial preoptic area, supra chiasmatic nucleus, lateral hypothalamus ventromedial hypothalamic nucleus, the arcuate nucleus, habenular nuclei, laterodorsal tegmental nucleus, pedunculopontine tegmental nucleus, locus coeruleus, nucleus of the solitary tract, the spinal trigeminal nucleus and the sub parabrachial nucleus [26] and have also presented evidence for high level expression of neuroglobin in brain areas involved in sleep/wake and food regulation [27,28] with intense neuroglobin expression in the hypothalmic supra-chiasmatic nucleus [29]. 

In the highly vascularised retina of mammals such as in the mouse the concentration of neuroglobin can achieve levels >100 μM; that is approximately 100 times the concentration in the brain [30]. Closer examination of the vascularised retina has identified selective neuroglobin expression within various cell types. In particular, high levels of neuroglobin have been reported in the photoreceptor inner segments, plexiform layers and ganglion cells [31]. A comparative study between mammalian vascularised and avascularised retinas has shown that in both cases neuroglobin is associated strongly with mitochondria [20]. In the dog retina immunohistochemistry has shown neuroglobin to be present in ganglion cells, inner and outer nuclear layers, inner and outer plexiform, photoreceptor inner segments and retinal pigment epithelium [32]. 

Thus some strong debate still exists concerning exactly which parts of the brain and the retina are the most significant in terms of neuroglobin expression. There is general consensus that neuroglobin, although wide spread in the brain, is preferentially expressed in certain regions. Much of the debate certainly arises from the use of neuroglobin antibodies. In general these have low titres, presumably dues to neuroglobin’s low antigenicity, as a consequence of its very high level of conservation, together with common cross reactivity with other proteins such as triose-phosphate isomerase [33]. In a recent study, using rigorously validated antibodies to neuroglobin and control measurements on neuroglobin null mice retinas, Hundahl *et al.* [34] have raised doubts about the validity of many previous antibody based studies. Furthermore, Hundahl *et al.* do a great service to the field by reminding us “When interpreting patterns of immunostaining it must be kept in mind that a staining pattern reports the location of the immunogen and as such it is devoid of any causal or mechanistic information. Consequently, a staining pattern should never be used as a major piece of evidence when drawing functional conclusions about the protein of interest” [34]. As in most cases the connection between measured RNA levels and protein concentration has not been established for neuroglobin. 

At the molecular level the control of neuroglobin expression is not yet fully understood. Early work in this area failed to find regulation by the classical HIF-1 α mechanism but suggested control by other response elements [35]. An analysis of the 5'-ﬂanking region of the human neuroglobin gene (NGB) identiﬁed a transcription start site (TSS) located −306 bp relative to the translation start site. The proximal promoter contained two GC-boxes located at −16 and +30 bp relative to the TSS which bound transcription factors Sp1 and Sp3. Two putative NRSE sites (−359 and −127 bp relative to the TSS) showed no inﬂuence on NGB tissue-speciﬁc expression. 5-aza-2′-deoxycytidine induced NGB expression, suggesting a potential role of DNA methylation in regulating NGB tissue-speciﬁc expression [36]. Very recent results have identified a core proximal promoter of 554 bp for the neuroglobin gene. This promoter has been shown to interact with NF-κB members p65, p50, cRe1, Egr1, and Sp1. κB3 appears to be a pivotal cis-element responsible for hypoxia induced neuroglobin expression. NFκB (p65) and Sp1 are also responsible for hypoxia induced upregulation of neuroglobin and HIF-1α was also suggested to be involved, but indirectly [37].

## 3. The Impact of Neuroglobin on Cultured Cells, Animal Models and Human Diseases

### 3.1. Cells

In cells in culture the expression of neuroglobin has been shown to be protective to a number of challenges. In PC12 cells expression of neuroglobin has been shown to protect from cell death induced by beta-amyloid and oxidative stress challenges [38,39]. Likewise, in the neuroblastoma cell line SH-SY5Y neuroglobin protects against oxidative stress [40], anoxia or oxygen and glucose deprivation [41] and specific challenge with the BH3 mimetic TW-37 [42]. Neuroglobin has also been shown to protect against NO toxicity in HN33 mouse hippocampal neuron x N18TG2 neuroblastoma cells [43].

Neuroglobin has been identified as being protective in cultured neurons. In a series of papers Wang and colleagues have shown neuroglobin overexpression in primary neurons following hypoxia/reoxygenation and oxygen/glucose deprivation and that these effects are correlated with mitochondrial function [44,45,46]. In other studies, cultured human neuronal cells have been shown to be protected from hydrogen peroxide insult when over-expressing neuroglobin [47]. Within all these studies the use of different cell lines, together with different insults, of different strength and duration, almost certainly account for the reported specific differences in outcomes. Never the less all of these studies clearly indicate the capacity of neuroglobin to ameliorate the impact of otherwise deadly challenges to cultured cells.

### 3.2. Animal Models

Very soon after neuroglobin was first discovered Greenberg and others, in a series of studies, conclusively showed that neuroglobin is protective in transfected animal models of ischaemic stroke [48,49,50,51]. More recently, studies have proceeded to transgenic animal models constitutively over-expressing neuroglobin and these have supported the previous findings that over expression of neuroglobin can typically reduce brain neuron cell death by approximately 30% in ischemic stroke [52,53]. In a very interesting, recent paper Hundahl *et a*l have reported on the impact of hypoxia on neuroglobin null mice [54]. They report no difference in cell survival between wild type and mutant mice, which is in contrast to the findings previously reported in virus mediated knock down mice. They, never the less, also report that in the neuroglobin null mice hypoxia induced an enhanced activation of HIF 1α and c-FOS. It is not yet clear whether this lack of difference is due to the use of different hypoxic models and focus on specific cell types by Hundahl *et al.* In the earlier studies the impact of neuroglobin was tested using a blood vessel ligation approach to simulate ischemic stroke whilst Hundahl *et al.* employed chronic exposure to 7% oxygen. Furthermore, Hundahl *et al.* specifically monitored orexin linked neurons whilst other workers have focused on global brain cell death. In a very recent paper Raida *et al.* [55] have shown that in neuroglobin-null mice a permanent cerebral artey occlusion (*cf* transient occlusion employed by Greenberg) after 24 h reduced the infarct size compared to the wild type. Thus in terms of the impact of the level of neuroglobin on infarct size, in animal models, conflicting data exist. The mild phenotype of the neuroglobin-null mice also raises queries as to the necessity for neuroglobin in brain.

The change in the expression level of neuroglobin in the brain of animal models in response to various insults has been investigated in a very large number of papers. In particular, an increase in expressed neuroglobin protein has been identified in brain in response to various levels of hypoxia in most reports [56,57,58,59,60,61]. However some have reported no change in the level of neuroglobin expression [62]. As in other studies it is not yet completely clear as to whether these reported differences in outcome really reflect the use of insults of different strength, nature and duration. Neuroglobin levels in brain have also been reported to increase following endotoxin, CO, arsenite, light exposure and traumatic injury [63,64,65,66,67,68]. In other circumstances neuroglobin has been shown to be up-regulated in ocular hypertension induced acute hypoxic-ischemic retinal injury and in the cochlea of rat pups exposed to mild CO poisoning [69,70]. An age related decline in the level of neuroglobin seen in rat models may relate to the loss of its protective role in age related degenerative disorders [71]. Although not normally seen in astrocytes there has been a recent report of the observation of neuroglobin in astrocytes [72] and reactive astrocytes in neuropathological conditions related to traumatic injury, cerebral malaria and autoimmune encephalitis [73].

### 3.3. Humans

Following on from their initial work on cells and model systems the groups of Greenberg and Wang have gone on to establish the significance of neuroglobin *in situ*ations of stroke, cerebral ischemia and intracerebral hemorrhage [74,75,76,77]. In one of the first population level studies involving neuroglobin no mutant forms of the coding region of neuroglobin was found, but polymorphism in the first intronic region of the neuroglobin gene was shown to be associated with a decreased risk of stroke in the Han Chinese population [78]. Increased expression of neuroglobin has been reported in a number of tumors. Neuroglobin is upregulated in both glioblastoma and astrocytoma [79,80]. In the case of non-small cell lung cancer neuroglobin is found to be upregulated in all samples but more often in squamous rather than adenocarcinoma [81]. In a wider ranging study it was found that neuroglobin was often upregulated and this lead the author to conclude that neuroglobin represents “a part of the defense repertoire that allow cancer cells to survive in hypoxic conditions” [82]. In other cases of brain dysfunction neuroglobin has been shown to attenuate beta-amyloid neurotoxicity and Alzheimer’s disease, showing genetic association and gene expression changes associated with Alzheimer’s dementia [83,84]. Neuroglobin is upregulated after traumatic brain injury in which functional outcomes show links to neuroglobin genetic polymorphisms [85,86].

In a series of papers Ostojic and co-workers have presented the distribution of neuroglobin in the normal retina [87,88,89]. The neuroglobin protein has been shown to be protective in retinal ischemia and also in the case of glaucoma [90,91,92].

## 4. Proposed Modes of Action of Neuroglobin

From the preceding sections it is clear that there is general consensus in the field that neuroglobin has the potential to protect cells and in particular brain neurons and retinal cells from insult initiated cell death and may have a significant role to play in the origin and progression of various disease states in humans. What is just as clear from these studies is that although many studies may give hints as to the mechanism whereby neuroglobin provides cell protection, no clear mechanism is obvious. This has led to some years of speculation as to potential protective mechanisms. Many of these speculations have been based on “guilt by association” style arguments which relate more to the site of expression rather than any direct investigations of molecular action. Furthermore, little attention seems to have been paid to the question as to whether neuroglobin might function by a common mechanism in all cell types, in response to the very varied cellular insults investigated. Many reviews and much speculation have appeared considering this problem of potential molecular mechanism [93,94,95,96,97,98,99,100,101,102,103]. In general the proposed mechanisms of action can be divided into those which consider the well characterized physiochemical interactions of neuroglobin with small gaseous molecules such as oxygen and nitric oxide, whilst others have focused on the unique interactions of neuroglobin with specific partner proteins. 

### 4.1. The Oxygen Model

Although normally considered six co-ordinate the heme contained in neuroglobin is in fact an equilibrium mixture between a predominant six co-ordinate structure and a minor five co-ordinate structure. Hence, in the reduced state neuroglobin can bind oxygen. By analogy with the function of myoglobin, the initial discovery of oxygen binding to neuroglobin led many to speculate that neuroglobin in neurons and retina might also function to store and facilitate the transport of oxygen to mitochondria in actively aerobic cells [99,104]. This proposal, in part, was argued on the basis of the, later proven incorrect, initial report of a high oxygen affinity for neuroglobin [12]. This original proposed function however has now been discarded on a number of counts. Modeling studies exclude the possibility of an assisted oxygen transport role, even at the high concentrations of neuroglobin reported in the retina [9]. In neurons the concentration of neuroglobin is much too low to facilitate oxygen delivery to mitochondria. The physiologically relevant oxygen affinity of neuroglobin has been shown to be nearly a magnitude lower than first reported and furthermore the oxygenated form of neuroglobin readily undergoes autoxidation to the ferric form [105]. Thus to maintain active oxygen binding would require a very active re-reduction system which, despite many efforts, has never been identified [106]. Brunori *et al.* initially suggested that neuroglobin might be re-reduced by AIF [107] but this has now been shown not to be the case [108]. In the absence of support for the suggestion that neuroglobin might be responsible for oxygen transport and based on the sites of expression in mice it has been suggested that neuroglobin expression is related to possible reactive oxygen species scavenging [109]. Although neuroglobin has been demonstrated to act as both an antioxidant and free radical scavenger its activity is lower than that of N-acetyl cysteine, vitamin c and glutathione and furthermore its reactions are slow [110]. Considering the relatively high levels of GSH and vitamin c in neurons it thus seems unlikely that neuroglobin has a major role in the scavenging of reactive oxygen species. 

### 4.2. The Nitric Oxide Model

As do many other heme proteins, neuroglobin shows a complex *in vitro* chemistry in its reactions with NO and nitrogen oxyanions. This complex chemistry is further complicated by its dependence on the presence or absence of oxygen and the initial redox state of the protein. Thus in the absence of oxygen the ferrous form of neuroglobin reversibly binds NO [111] and reacts with nitrite ion to give the ferric NO complex and NO [112,113]. It has recently been suggested that neuroglobin may have a biological role as a nitrite reductase [114]. However, even at elevated, non-physiological levels of neuroglobin and nitrite the reaction is slow (k = 0.2 M^−1^ s^−1^, t_1/2_ = approx. 10 min) even under totally anaerobic conditions. Under physiologically relevant levels of oxygen it is difficult to see how the nitrite reductase reaction might be important given that the competing reaction of the ferrous neuroglobin with oxygen is extremely rapid (k = 1.7 × 10^8^ M^−1^ s^−1^, rate limited at the histidine off rate of approximately 1.0 s^−1^) [14]. In the presence of oxygen neuroglobin forms an oxygenated complex which reacts with NO yielding the ferric protein and nitrate ions [13]. This has been suggested to be a major role for neuroglobin in NO scavenging. However, more recent studies have shown that neuroglobin is no more effective in this reaction than myoglobin and thus raises the question—if this were the role for neuroglobin then why is it present at low concentrations and why is it so conserved? [114]. The ferrous NO complex reacts with peroxynitrite to give the ferric form of the protein but does not produce the ferryl product in the presence of either hydrogen peroxide or peroxynitrite [115,116]. When neuroglobin is in the ferric form addition of excess NO yields the ferrous NO complex [114]. Although neuroglobin shows this rich *in vitro* chemistry in its reactions with NO and nitrogen oxyanions, which is further complicated by reactions of many intermediates with oxygen, it is not at all clear as to the significance, *in vivo*, of this potential reactivity. In particular many of the reactions would require re-reduction of neuroglobin to be of significance and, as outlined above, no re-reducing system has yet been identified. As far as this author is aware there have been no experimental studies reported on the direct reactions of neuroglobin with NO *in vivo*. As such the potential significance of neuroglobin in NO homeostasis and scavenging remains to be proven.

### 4.3. Protein-Protein Interaction Models

Neuroglobin not only interacts with small gaseous ligands such as oxygen and nitric oxide, it also has been reported to interact with a number of other proteins. Two recent large scale investigations have been reported which investigate interactions between neuroglobin and other proteins. In one, affinity purification/MS analysis of the cell lysates of HN33 cells, under aerobic and hypoxic conditions, was used to identify interaction partners. This study indicated that neuroglobin interacts with antioxidant related proteins cyt c, cyt c1, AIF, Thio and Prdx3,4 and 6 and the Alzheimer’s associated proteins Thop1, Zpr1,DJ-1 and L1 [117]. In another study, using yeast two hybrid methods to study the interactions of neuroglobin with proteins encoded in a mouse cDNA library, 10 partner proteins were identified 6 of which were also present in an immune-precipitation study of proteins obtained from primary cultures of mouse cortical neurons. These six proteins were Atp1b1, Cyc1, Ubc, Dvl1, Etfa and VDAC [118]. In a series of papers, Wakasugi and colleagues have made a strong case for the action of neuroglobin as a heterotrimeric Gα protein guanine nucleotide dissociation inhibitor. In this case the ferric form of neuroglobin binds to the Gα subunit of the GCPR preventing GTP/GDP exchange, preventing activation of the Gα subunit but favouring Gβγ pathways, which amongst other things activate PI3K [119,120,121,122,123,124,125]. It should be noted that the ferrous form of neuroglobin does not show such an interaction. Neuroglobin has also been shown to interact with the prion protein and the plaque associated tau protein, although the binding constant for tau (10μM) would suggest that the interaction is unlikely in neurons [126,127,128]. The protein 14-3-3, which plays an important role in apoptosis by phosphorylating BAD, also interact with neuroglobin [129,130] and there is some evidence that the consequence of this interaction is the phosphorylation of neuroglobin, which alters the heme pocket co-ordination [131]. Ferrous neuroglobin has also been shown to react in one of the fastest known protein-protein interactions with ferric cytochrome c, via the formation of an intermediate complex, followed by rapid electron transfer. [132,133].

In summary of the experimental findings relating to neuroglobin:

(1) Neuroglobin is found in some brain neurons, retina and some endocrine cells.
(2) The presence of neuroglobin protects cultured cells from various challenges such as hypoxia and NO.
(3) In animal models over-expression of neuroglobin protects against stroke and brain injury.
(4) In humans neuroglobin appears up regulated following stroke, Alzheimer’s and glaucoma and is over expressed in some tumors.
(5) Neuroglobin appears to interact with specific proteins such as cytochrome c, GCPR, 14-3-3.

As outlined above, various suggestions have been made concerning possible mechanisms of action of neuroglobin. In each case these mechanisms seem to have been concerned primarily with the role of neuroglobin in regards to a particular type of challenge. One proposal has however been made which tries to rationalize the normal site of expression, the response to various challenges and the known reactivity of neuroglobin.

## 5. Neuroglobin and Apoptosis

Based on the initial findings of the reactivity of neuroglobin with cytochrome c [132,133] it was hypothesized that perhaps neuroglobin’s actions, in protecting cells from cell death, in various situations, could be explained by consideration of the process of apoptosis, as all the cellular insults, be it in cultured cells, animal models or human trauma or diseases have been shown to involve cell death via the process of apoptosis [134,135,136]. Hypoxia and NO induce apoptosis via disruption of mitochondrial function [137]. This proposal would be consistent with the sites of neuroglobin expression. Brain neurons and retinal rod cells are unique in that they cannot be replaced by cellular replication and hence need to be particularly well protected from unintentional apoptosis [138]. Tumor cell survival often involves suppression of the normal apoptotic processes. Most of the protein interactions with neuroglobin, so far identified, involve proteins which have well defined roles in the apoptotic process. 

**Figure 2:**
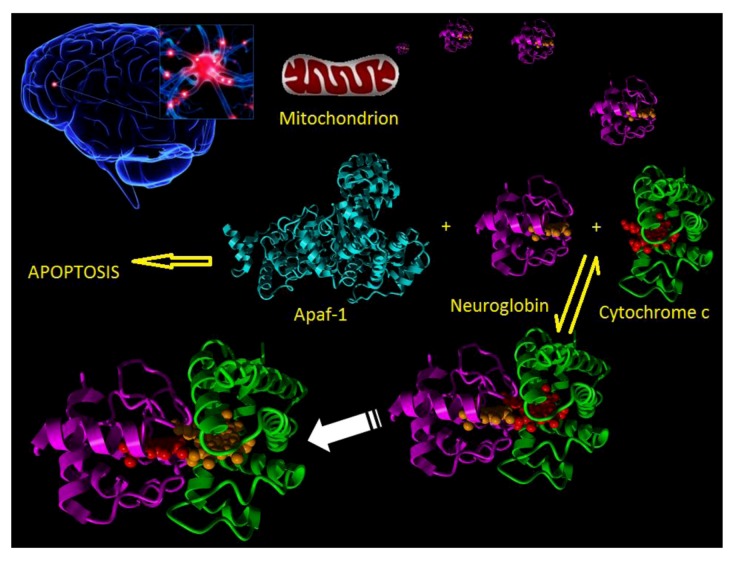
Cartoon showing the molecular events following a stroke. Neuron damage (top left) leads to mitochondrial release of cytochrome c (magenta) into the cytosol. In the cytosol cytochrome c can bind to either Apaf-1 (cyan) or neuroglobin (green). Binding to Apaf-1 leads to apoptosome formation and apoptotic cell death. Binding of cytochrome c to neuroglobin and subsequent reduction prevents cell death.

In the normal course of events when a neuron experiences an appropriate stressor then the intrinsic apoptotic pathway is activated. In this process the stressor disturbs the normal balance between pro and anti-apoptotic interactions between such proteins as BAD, BAX, BID and Bcl2 family members such as to induce the formation, in the mitochondrial membrane, of the permeability pore. This then leads to the release of mitochondrial cytochrome c into the cytosol. Released cytochrome c interacts with Apaf-1 to produce the macromolecular apoptosome complex which goes on to activate caspase 9 and finally the execution caspases lead to final destruction of the cell (Figure 2). 

**Figure 3:**
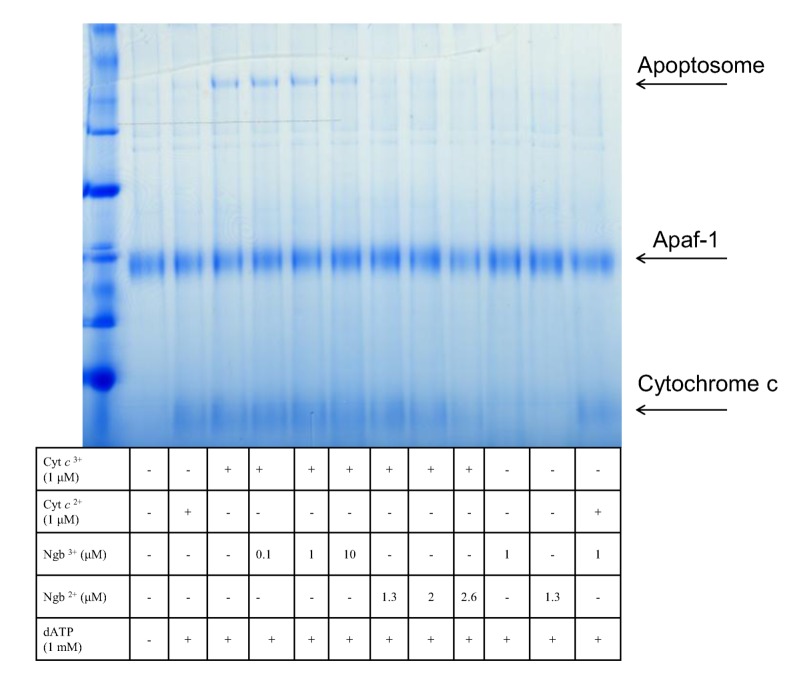
Native blue gel showing Apaf-1 binding to oxidized cytochrome c in the presence of ATP to give the apoptosome complex.

The apoptotic model for the mechanism of action of neuroglobin envisaged the interruption of this process by the sequestering of the released cytochrome c and its reduction to the non-apoptotic ferric form. Previous work had highlighted the necessity of the ferric form of cytochrome c in order to propagate the apoptotic pathway [139]. In unpublished work we have established that the requirement for the ferric form of cytochrome c relates to the fact that only this redox state is capable of inducing apoptosome formation with Apaf-1 (see Figure 3). 

This hypothesis, relating neuroglobin interaction to apoptosis, required validation. In a series of papers Brittain and colleagues have investigated the anti-apoptotic activity of neuroglobin in cultured cells. It has now been shown that in cultured SH-SY5Y cells the presence of neuroglobin lowers the susceptibility of the cell to challenge by apoptotic specific BH3 mimetics HA14 and TW-37 and serves to preserve the mitochondrial transmembrane potential [140,141,142]. Associated construction of an experimentally validated systems level, stochastic model of the apoptotic process has allowed the investigators to explore conditions not easily amenable to laboratory experiment. These investigations clearly indicate that the effect of neuroglobin within cells is very concentration depended and, most importantly, that the presence of neuroglobin does not prevent apoptosis but rather raises the trigger level of stressor which needs to be achieved before the process of apoptosis is committed to. This last point may well account for the somewhat variable results reported in early studies using variable duration and intensity of cellular stressors. Thus, this model appears to account for much of the literature findings by simply recognizing the direct interaction of neuroglobin with cytochrome c during activation of the intrinsic apoptotic pathway can prevent activation of the terminal caspase proteases responsible for cell destruction. Implicit in this model is not only the sequestration and reduction of cytochrome c but also the production of ferric neuroglobin, both of which further explain other observed, experimental results. Cytochrome c not only interacts with Apaf-1 to produce the apoptosome, it is also known to have a major role in the amplification of the apoptotic process, so necessary for the full committal to cell death. A major controller of cytosolic calcium concentration is the auto-regulated endoplasmic reticulum associated calcium channel. This channel maintains the cytosolic calcium concentration to the nM region by a combination of IP3 stimulated release and an auto-regulated calcium feedback mechanism. The auto-inhibition of calcium release is relieved in the presence of cytochrome c [143,144]. Thus, in the absence of cytochrome c sequestration, cells, once set onto the apoptotic path by release of cytochrome c from one mitochondrion, will experience a rapid rise in cytosolic calcium concentration. Once calcium reaches a level of 0.8μM, calcium ions themselves are sufficient to precipitate apoptotic responses in other mitochondria and hence initiate the avalanche characteristics of apoptosis. In the presence of neuroglobin the level of cytochrome release must achieve a significantly higher value before the calcium linked cascade can operate. That neuroglobin can indeed limit calcium levels, has been shown directly by X-ray fluorescence [145,146]. Considering the normal resting calcium level in a cell, the increase in calcium associated with calcium transients produced during normal functioning of neurons and retinal rod cells and the trigger level required to activate apoptosis, it is tempting to suggest this explains why glutamatergic neurons in brain and rod cells in the eye contain neuroglobin. Under normal operating conditions during neuron firing or rod cell activation the calcium level reaches values very close to those known to precipitate the apoptotic cascade. Minor random statistical effects could then in the absence of neuroglobin lead to the undesired death of these non-replicating cells with consequent loss of function of the brain and eye. In the presence of the appropriate level of neuroglobin the threshold for apoptotic activation would be raised above that which might otherwise lead to accidental apoptotic cell death whilst not preventing appropriate initiation of cell death, following strong stressor activation of the intrinsic apoptotic pathway.

**Figure 4:**
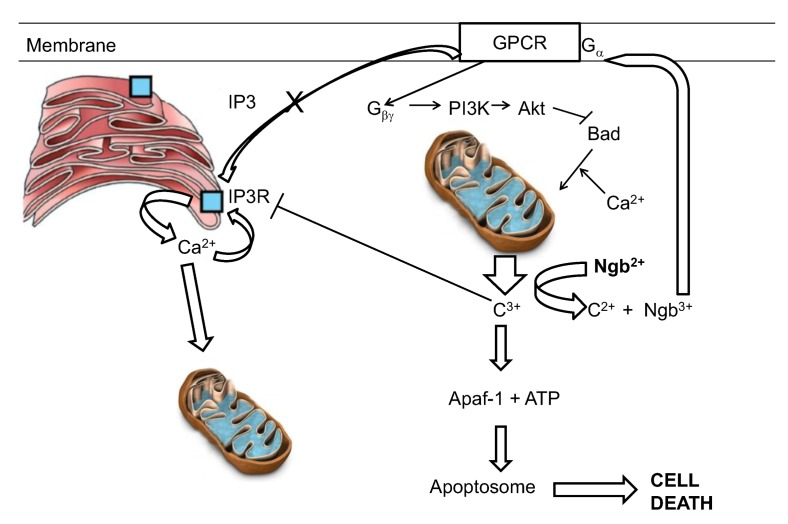
A hypothetical interpretation of the know reactivities of neuroglobin in the suppression of apoptotic activity. C3+ and c2+ represent the oxidized and reduced forms of cytochrome c respectively.

The reaction of ferrous neuroglobin with cytochrome c will yield ferric neuroglobin, which, as described above, has been shown by Wakasugi to be a potent inhibitor of G_α_ of the GCPR. Inhibition of the Gα subunit will prevent production of IP3 and hence reduce calcium uptake into the ER but will also free Gβγ to activate PI3K and Akt, both anti-apoptotic protein [128,147] (Figure 4). 

The final aspect of this model relates to the redox status of the cytosolic neuroglobin. Neuroglobin, as pointed out above, binds oxygen and can then undergo autoxidation. Thus, in a live cell the relative levels of reductant and oxygen will set up a futile cycle in which neuroglobin undergoes a cycle of reduction, oxygen binding, autoxidation and re-reduction. The steady state level of the anti-apoptotic ferrous form of neuroglobin will then be controlled by the reductant/oxygen ration within the cell [148]. This cycle has been mathematically modeled using experimentally determined autoxidation and reduction rates [148,149]. The outcome of these investigations clearly suggests that under normoxic conditions the level of reduced neuroglobin is relatively low. However under conditions of decreasing oxygen concentration, as might be seen following stroke, the system has the capacity to rapidly convert the ferric neuroglobin to ferrous neuroglobin, so bolstering the cells capacity to withstand apoptotic challenge. It should be made clear however that the redox cycling of neuroglobin, as envisaged in the model above, occurs at a rate much slower than the rate of reaction of neuroglobin with cytochrome c. The redox cycle sets the “steady state” level of reduced neuroglobin instantly available to interact stoichiometrically with cytochrome c and thus defines the level of insult (in terms of the amount of cytochrome c released) which can be tolerated by the cell and is not envisaged to undergo any redox cycling on the time scale of the interaction involving reaction with cytochrome c. All in all, although not fully proven, this model (see Figure 4) would at the present time appear the best able to account for the known functions and interactions seen for neuroglobin. This model however gives no insight into the major remaining conundrum—if the role of neuroglobin is to protect the cell from unwanted, accidental apoptosis then why is its distribution in brain confined to just a few well defined areas?

## 6. Potential Neuroglobin Therapies

As soon as it was realized that neuroglobin can prevent cell death, potential therapeutic interventions have been explored. In early studies, using a fusion protein between neuroglobin and the cell penetrating HIV transcriptional transactivator peptide TAT, it was found that treatment of isolated islet cells prolonged their viability and may well prove useful for islet transplants in cases of type I diabetes [150]. Translocation and neuroprotection in primary cultured cortical neurons by the TAT-neuroglobin fusion protein was also reported [151]. However, in a cellular stroke model, based on oxygen /glucose deprivation in CHO and SHY5Y cells, no protective effect was evident for the TAT-neuroglobin fusion protein [152]. In a mouse model of focal cerebral ischemia it was shown that the TAT-neuroglobin construct crossed the blood-brain barrier and protected against brain damage if delivered before the insult [153]. The work on using TAT-neuroglobin fusions to protect cells has recently been reviewed [154]. In a fascinating discovery, Wakasugi and colleagues have shown that replacement of the first exonic segment of human neuroglobin with that of the zebra fish produces a fully functional protein which has cell permeability [155,156].

In a very recent *in vivo* study it was demonstrated that lentivirus mediated neuroglobin gene delivery to the spinal cords of rabbits suffering spinal cord injury reduced both secondary damage and improved the final outcomes [157].

Small molecule treatment based on upregulation of endogenous neuroglobin levels has also been investigated. In HN33 cells in culture it has been shown that deferoxamin, cinnamic acid and valproic acid all lead to an increase in the cellular concentration of neuroglobin [158]. 17β estradiol has also shown promise in the upregulation of neuroglobin [159] and in fact has been known to be protective against stroke in post-menopausal women in for some time [160]. However it is also known that estradiol is potentially carcinogenic [161,162]. The area of targeting endogenous neuroglobin for the protection against stroke and neurodegenerative disorders has recently been reviewed [163].

## 7. Conclusions

Much evidence has been gathered indicating the cell protective function of neuroglobin both in neurons and retinal rod cells in culture, animal models and humans. This cell protection is apparent in oxygen/glucose deprivation as well as H_2_O_2_ and amyloid challenge. In humans data is accumulating which indicates protection in such circumstances as stroke, Alzheimers and glaucoma. The mode of cell protection has recently been shown to be in the main, if not completely, due to the capacity of neuroglobin to intercept the apoptotic intermediate cytochrome c, released from mitochondria. Both mathematical modeling and experimental studies indicate that the presence of neuroglobin, rather than preventing apoptosis, raises the level of insult necessary to initiate the onset of the apoptotic cascade. Attention has recently turned to the potential of neuroglobin in therapeutic situations.
